# Integrating scFv into xMAP Assays for the Detection of Marine Toxins

**DOI:** 10.3390/toxins8110346

**Published:** 2016-11-21

**Authors:** Lisa C. Shriver-Lake, Jinny L. Liu, P. Audrey Brozozog Lee, Ellen R. Goldman, Richard Dietrich, Erwin Märtlbauer, George P. Anderson

**Affiliations:** 1 U.S. Naval Research Laboratory, Center for Biomolecular Science and Engineering, 4555 Overlook Ave SW, Washington, DC 20375, USA; lisa.shriverlake@nrl.navy.mil (L.C.S.-L.); jinny.liu@nrl.navy.mil (J.L.L.); ellen.goldman@nrl.navy.mil (E.R.G.); 2 Nova Research Inc., 1900 Elkin St., Suite 230, Alexandria, VA 22308, USA; plee142@su.edu; 3 Chair of Hygiene and Technology of Milk, LMU München, Schönleutnerstraße 8/219, 85764 Oberschleißheim, Germany; r.dietrich@mh.vetmed.uni-muenchen.de (R.D.); e.maertlbauer@mh.vetmed.uni-muenchen.de (E.M.)

**Keywords:** saxitoxin, domoic acid, single chain antibody, xMAP

## Abstract

Marine toxins, such as saxitoxin and domoic acid are associated with algae blooms and can bioaccumulate in shell fish which present both health and economic concerns. The ability to detect the presence of toxin is paramount for the administration of the correct supportive care in case of intoxication; environmental monitoring to detect the presence of toxin is also important for prevention of intoxication. Immunoassays are one tool that has successfully been applied to the detection of marine toxins. Herein, we had the variable regions of two saxitoxin binding monoclonal antibodies sequenced and used the information to produce recombinant constructs that consist of linked heavy and light variable domains that make up the binding domains of the antibodies (scFv). Recombinantly produced binding elements such as scFv provide an alternative to traditional antibodies and serve to “preserve” monoclonal antibodies as they can be easily recreated from their sequence data. In this paper, we combined the anti-saxitoxin scFv developed here with a previously developed anti-domoic acid scFv and demonstrated their utility in a microsphere-based competitive immunoassay format. In addition to detection in buffer, we demonstrated equivalent sensitivity in oyster and scallop matrices. The potential for multiplexed detection using scFvs in this immunoassay format is demonstrated.

## 1. Introduction

Food poisoning from naturally occurring marine toxins is a worldwide public health issue, and it poses economic issues/concerns for the food industry. Marine toxins, such as saxitoxin (STX) and domoic acid (DA), are associated with algae blooms and can bioaccumulate in shellfish and herbivorous fishes causing food poisoning [1,2]. The frequency of STX and DA producing algae blooms is on the rise [3], possibly due to climate change, leading to increasing potential for adverse environmental, economic, and health implications.

STXs, produced by dinoflagellates, are the most well studied cause of paralytic shellfish poisoning (PSP) which is also the most common and lethal form of marine toxin poisoning [2,4]. PSP results in paralysis of muscles throughout the body and at higher concentrations can cause death. STX, is a heat stable toxin whose primary target is the voltage-gated sodium channels in the nerve and muscle cells of the body affecting the gastrointestinal and neurological systems [4,5]. Because of its potency, STX has been identified as a potential biothreat agent and is regulated as a select agent by the U.S. Center for Disease Control (CDC).

DA is a representative of toxins that cause amnesic shellfish poisoning (ASP). Besides typical food poisoning symptoms, short term memory loss, confusion, and disorientation are observed in ASP. Although higher levels of DA are needed for intoxication, it is a heat stable toxin that can cause kidney damage at levels several orders of magnitude lower than those that cause neurological symptoms [5,6]. It acts by stimulating the glutamate receptors.

There is no known antidote for either STX or DA poisoning; therefore, the ability to detect the presence of toxin is vital for the early administration of the correct supportive care [7]. Detection of these and related toxins are also of importance for environmental monitoring to limit use of shellfish from that region. Until the last decade, the mouse bioassay (MBA) was the only approved method for the detection of marine toxins. The MBA provides toxicity information about the food sample but not toxin identification. Moral and ethical issues with this method (animal usage) have led to the development of other methods. Liquid chromatography such as HPLC (with fluorometric detection for STX), LC-UV for DA, LC-MS and UPLC-MS/MS [2,4,8,9], are methods that are able to detect and identify variants of the toxins. HPLC-FD and LC-UV have been validated for use in the EU. Surface Enhanced Raman Scattering (SERS) has also been demonstrated [9,10]. While these methods have the specificity, they require extensive sample preparation, expensive equipment, highly trained personnel, and the availability of analytical standards. In addition, interferences from complex matrices have been observed.

Alternatively, biologically-based assays have been developed that can monitor toxicity similar to the MBA and, thereby, provide for rapid screening of samples. Cell-based sensors have been developed for PSP toxins, which utilize only cells instead of animals [11,12,13]. In 2012, Van Dolan et al. described a receptor-based assay that was able to detect PSP toxins near the regulatory limits [4,14]. Even though cells and receptors have been employed to identify marine toxins and their toxicity, most bio-based methods employ antibodies as a rapid screening tool. Several different immunoassays have successfully been applied to the detection of marine toxins that use either polyclonal (pAbs) or monoclonal antibodies (mAbs) against STX and DA [15,16,17,18,19]. Antibodies have been used in many different detection formats such as: enzyme-linked immunosorbent assays (ELISAs) [20,21], surface plasmon resonance (SPR) [22,23,24,25], fluorescence/chemiluminescence-based microarray assays [26,27] flow cytometry [28,29], and lab-on-a-chip using fluorescence and magnetic particles [30]. Recently, two groups have been developing lateral flow immunoassays (LFIs) for the detection of PSP toxins and DA as rapid, low tech screening assays [31,32,33]. Campbell et al. reviews biological toxin binders in detail in their 2011 paper [34].

In the last few years, concerns have grown over the reliability and reproducibility of standard antibodies. A. Bradbury wrote an article in Nature describing the issues with using traditional monoclonal and polyclonal antibodies [35]. These issues include availability of antibody (polyclonals -batch-to-batch variability), use of animals, and for monoclonals, the death of hybridoma cell lines. A viable alternative is recombinant antibodies including single-chain variable fragment (scFv), and single domain antibodies (sdAb) [36]. Recombinant constructs (scFv) that consist of linked heavy and light variable domains that make up the binding domains of conventional antibodies have been produced for the sensitive detection of DA, as well as a number of other biotoxins [37,38]. Recombinantly produced binding elements serve to “preserve” mAbs as they can be easily recreated from their sequence information, thereby reducing variability and eliminating issues with loss of cell lines. An additional advantage of scFv over mAbs is the ability to produce reagent recombinantly in *E. coli* and the potential to produce fusion constructs with enhanced utility that can potentially be tailored to particular sensor systems [38,39,40,41]. While not universal, improvements in stability, affinity, and diversity have been observed in scFvs, for example, improvement in both stability and affinity was demonstrated by McConell et al. [42].

Herein, we demonstrate recombinantly produced antibody recognition domains, scFvs, in a microsphere-based competitive immunoassay for the detection of STX and DA. This work utilized the previously described anti-DA binding fragment [37] in conjunction with an anti-STX binding domain that was synthesized from the sequence of an anti-STX mAb [15]. In addition to detection in buffer, we show the utility of the assay in shellfish matrices.

## 2. Results and Discussion

### 2.1. Sequencing and Evaluation of Anti-STX mAbs for scFv Production

**Figure 1:**
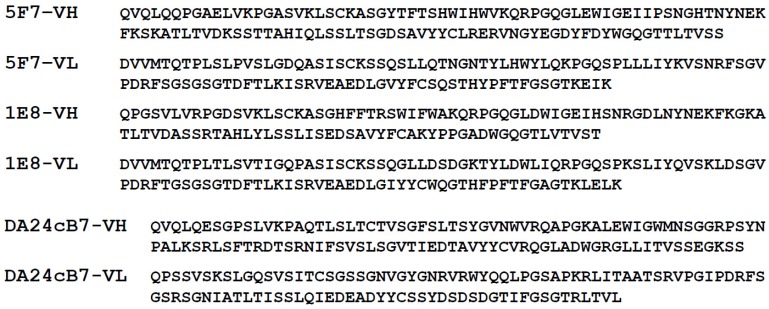
Sequence of the variable heavy chain (VH) and variable light chain (VL) regions of anti-saxiton (STX) monoclonal antibodies (mAbs) 5F7 and 1E8, and anti- domoic acid (DA) single-chain variable fragment (scFv) DA24cB7.

**Figure 2:**
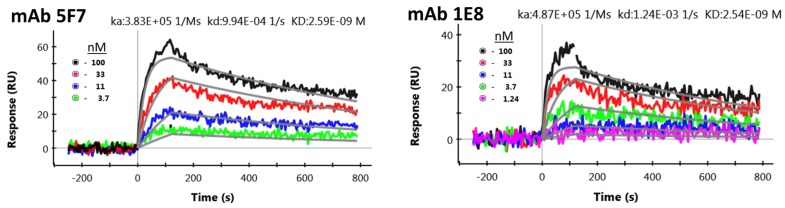
Surface plasmon resonance evaluation of anti-STX mAbs. The binding affinities of anti-STX mAbs, 5F7 and 1E8 were each evaluated on a surface with immobilized STX-HuIgG. Each mAb was tested simultaneously at six concentrations with an association time of 90 s and a dissociation time of 600 s. See Experimental Section for additional details.

The hybridoma supernatants and cell lines for the sequencing of anti-STX mAbs 5F7 and 1E8 were developed at Ludwig-Maximilians-Universität Munich (LMU) [15]. We contracted with Genscript (Piscataway, NJ, USA) to have the variable regions of the mAbs sequenced as well as for the production of each mAb for evaluation. Sequencing showed that 5F7 and 1E8’s sequences were unique (Figure 1). The mAbs were evaluated by surface plasmon resonance (SPR) for their ability to bind to a STX-IgG-antigen (Figure 2). STX was coupled to an irrelevant human IgG (HuIgG); the binding kinetics of mAbs 5F7 and 1E8 were observed to be ~2.6 and 2.5 nM, respectively.

**Figure 3:**
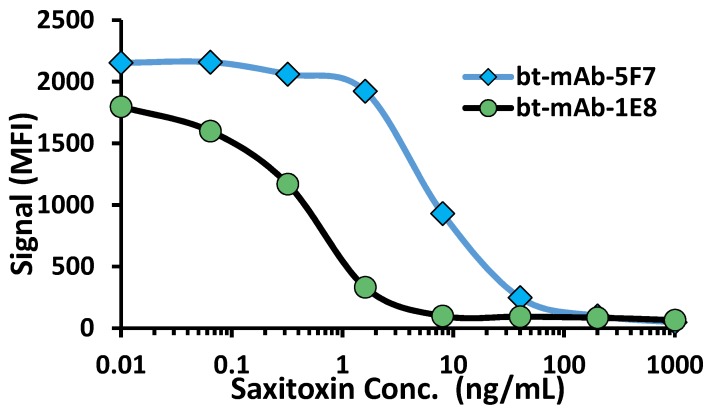
MAGPIX xMAP STX competitive immunoassay using mAbs. Each mAb was biotinylated and tested at 1 µg/mL in a competitive assay using STX-HuIgG coated MagPlex beads as described in the experimental section. Additional control bead sets are not shown. The graph is compiled from separate STX dose response assays for each of the mAbs.

The mAbs were also shown to function in xMAP assays on the MAGPIX instrument. First, each mAb was biotinylated and the dose response direct binding to STX-coated microspheres was evaluated to determine an appropriate concentration to use for a competitive assay (not shown). Next, the two mAbs were shown to function in a competitive format for the detection of STX (Figure 3). The results were very similar to those observed previously in a competitive ELISA assay [15], with 1E8 in this format appearing to have a higher affinity for STX and providing a better limit of detection.

The use of IgG for conjugation of the STX was due to the need to have a glycosylated molecule onto which the STX can be attached. Conjugate preparation followed a procedure that couples through the carbohydrate of the antibody to amines on the antigen paralleling the chemistry originally utilized for the immunogen used to prepare the mAbs [15]. STX was conjugated to IgG through a periodate chemistry that specifically activates the carbohydrate residues (described in Section 4.6); once activated, the aldehyde formed is highly reactive toward nucleophiles, especially primary amines. The highly negative pI of glucose oxidase (GO) used in the mAb production makes it less suitable for our application as it does not immobilize well onto the microspheres. The long linker length between the protein and the immobilized STX provided by carbohydrate side chains may contribute to the superiority of this chemistry for formation of both immunogen and toxin analog.

### 2.2. Production of scFv Targeting STX and DA

The protein sequences of the variable heavy and light domains from anti-STX mAbs 5F7 and 1E8 were synthesized with the variable heavy domain as a NcoI-NotI fragment and the light chain with flanking BamH1 and XhoI sites with a 26 amino acid long linker (containing the Not I and BamH1 sites) joining the two domains. The DNA for expressing the scFv was inserted, using NcoI-XhoI, to the pET22b(+) expression vector for periplasmic protein production.

Protein yields of ~1–2 mg/L were obtained from the 5F7 scFv, however, although we tried several times, we were unable to purify protein from the 1E8 scFv. We chose to focus on developing an anti-STX xMAP assay utilizing the 5F7 scFv. In the future, strategies such as expression with chaperones, fusion with a constant domain, or CDR grafting could be implemented for production of the 1E8 scFv [42,43,44].

For detection of DA, we were kindly provided with the protein sequence of the anti-DA scFv DA24cB7 (Figure 1) by Dr. Marian Kane [37]. We had the gene synthesized with flanking NcoI and XhoI restriction sites for cloning and a 15 amino acid linker between the variable heavy and light chains. The scFv was cloned into pET22b(+) for expression and typically yielded ~5–10 mg/L.

### 2.3. Evaluation of scFv Targeting STX and DA

**Figure 4:**
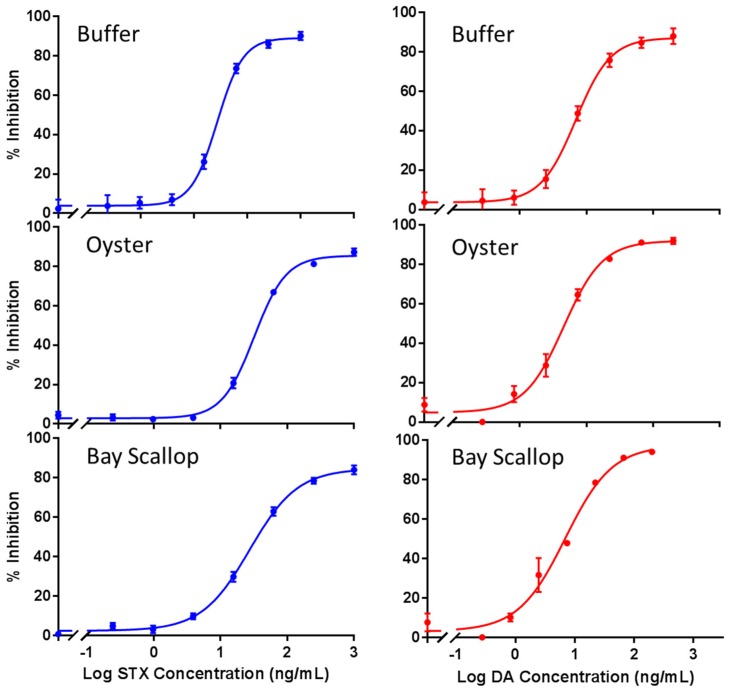
Dose response curves for STX and DA. The left side is % inhibition of the dose responses for STX in buffer (**top**), oysters (**middle**), and bay scallops (**bottom**). The right side is % inhibition of the dose response curves for DA in buffer (**top**), oysters (**middle**), and bay scallops (**bottom**). Data shown are from four to six replicates plus their SEMs.

**Figure 5:**
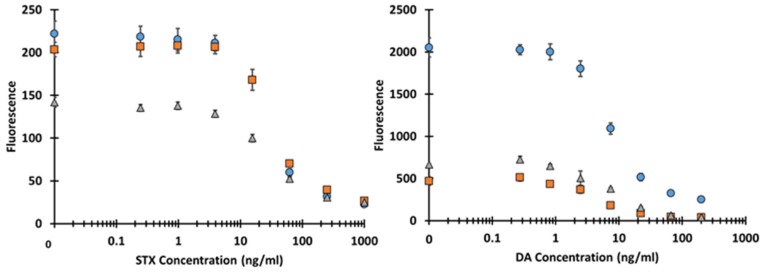
Average fluorescence dose response curves for STX and DA. The left side is STX dose responses in buffer (**blue circles**), oysters (**orange squares**), and bay scallops (**grey triangles**) and the right is DA. Each point represents the average of both bead sets for three experiments plus their SEMs.

Like monoclonal and polyclonal antibodies, scFvs have been incorporated into ELISAs, SPR, and LFIs [38]. Although the ELISA format affords amplification through the action of an enzyme, it is not easily multiplexed to examine multiple toxins in one well. The Luminex xMAP methodology consists of assays performed on color coded beads that can be highly multiplexed. Both sandwich and competitive format immunoassays have been demonstrated using the Luminex system [17,45,46]. In this article, an xMAP assay monitored using a MAGPIX system for the individual detection of STX and DA utilizing scFv recognition elements was demonstrated, Figure 4, Figure 5, and Figure S1.

To demonstrate the ability of the biotinylated-scFv to detect either STX or DA, competitive immunoassays with toxin-coated beads were performed in buffer and commonly contaminated shellfish matrices. For sample preparation of the seafood materials, a simple extraction protocol was utilized, however, more complex extraction methodologies that are likely more efficient have been described [27,47]. As shown in Figure 4, the scFvs were able to detect STX or DA in all three matrices in a dose dependent manner. Beads sets in which STX was coupled to human IgG (HuIgG) or rabbit IgG (RbIgG) showed similar dose response curves while the curves for DA microspheres which were prepared identically gave identical responses (Figure S1). The IC_50_, IC_10_, IC_90_, Min, and Max were determined (Table 1) by fitting the dose response curves using SigmaPlot 12.0 (Systat Software, San Jose, CA, USA) with a four-parameter logistic equation, Figure 4. Averages of the raw data are shown in Figure 5. Cross-reactivity studies for closely related compounds were not performed for the anti-STX scFv tested here, but have previously been examined for its parental mAb [15]. The IC_10_, which is the lowest concentration that these assays can be presumed to detect, was in the low ng/mL for both STX and DA. These limits are sufficient for most monitoring needs, but were not as sensitive as have been reported for a number of techniques. The higher IC_10_ could be for a number of reasons. One is that the concentration of antibody used in the competition assay will be inversely proportional to the IC_10_ observed; this allows for a more robust assay as a low concentration of antibody can lead to greater variances in the signal generated. It is likely, that with additional optimization of the assay reagents, improvements in sensitivity could be realized. Nonetheless, for our purposes of demonstrating these scFv immunoreagents in an xMAP assay format, these sensitivities were respectable.

As this work only examined standard curves for the detection of STX and DA in the various oyster and scallop extracts in comparison to a standard curve generated in buffer, extraction efficiencies were not calculated. However, it is possible to evaluate the matrix effect’s impact on the ability to discriminate the toxins. By comparing the magnitude of signal response in each matrix to that in buffer and the corresponding maximum signal divided by the minimum signal (signal-to-noise, S/N), one can estimate how the matrices affect the assays, see Table 2. The results of this analysis on the raw data shows that for the STX assay the response is slightly reduced for the oyster matrix and degraded ~50% for the scallop matrix, but the ability to discriminate that signal based on the ratio of S/N shows little change. For the DA assay, the signal strength is dramatically reduced in the matrices, especially for the oyster assay, which was reduced to ~1/4 the buffer signal levels, however, when comparing the S/N for the DA assays, the ratio actually improved in the extracts, possibly due to a reduction in nonspecific binding. Thus, it appears that the ability to discriminate STX and DA in the seafood extracts is not severely degraded, suggesting this minimal extraction protocol may warrant further evaluation to determine its efficiency as compared to others [27,47].

### 2.4. Combined STX and DA Assays

Preliminary efforts to combine the two assays were also undertaken. A combined assay for STX and DA was performed by mixing beads specific for STX and DA as well as both corresponding biotinylated scFvs. Figure S2 shows the results for the spiked STX or DA samples. While there is an increase in the inhibition for the STX microspheres in the DA spiked samples, there is a clear difference in signal at the higher concentration of free DA. The corresponding assay with STX had larger cross-reactivity issues. The cross-reactivity could be due to non-specific binding of the biotinylated scFvs to the microspheres in the absence of antigen. Further work would be required to develop or optimize an assay with less cross-reactivity, including blocking the beads after toxin immobilization or adjusting the buffers and wash buffers utilized to minimize this unwanted condition.

## 3. Conclusions

The ability to overcome disadvantages of pAbs and mAbs (e.g., availability and animal usage) by using recombinant constructs such as scFvs is the way of the future. In this study, we demonstrated the use of scFvs for the detection of two marine toxins: STX and DA. We employed these scFvs in competitive assays using the xMAP fluid array technology, obtaining limits of detection that approached those obtained with other antibodies, although further optimization is warranted. We demonstrated detection in both buffer and spiked food matrices with minimal sample preparation. Using the sequence information provided herein, these recognition molecules (scFvs) are now available to other researchers for incorporation into their immunoassay platforms as an alternative to traditional antibodies, and they could also be further engineered to include biotin binding domains or signal transduction domains (i.e., alkaline phosphatase) to further enhance their utility.

## 4. Experimental Section

### 4.1. Materials

MagPlex microspheres and MAGPIX Instrument were from Luminex Corporation (Austin, TX, USA). Streptavidin–R phycoerthryin (SA-PE) was purchased from Columbia Biosciences (Frederick, MD, USA) and the biotinylated anti-streptavidin (anti-SA) was obtained from Vector Laboratories (Burlingame, CA, USA). Phosphate-buffered saline with Tween and BSA (PBSTB) were prepared from phosphate-buffered saline with 0.05% Tween-20 (PBST) packets and 0.1% bovine serum albumin (BSA) from Sigma-Aldrich Chemical Co. (St. Louis, MO, USA). The saxitoxin dihydrochloride, 100 µg/mL in 20% ethanol-water solution (pH 3), was the kind gift of Dr. Sherwood Hall, US FDA, College Park, MD, USA. Domoic acid was purchased from Calbiochem EMD Biosciences (San Diego, CA, USA).

### 4.2. scFv Construction and Protein Production

The genes coding for the variable heavy and light domains from anti-STX mAbs 5F7 and 1E8 were synthesized with codons optimized for expression in *E. coli* (GenScript, Piscataway, NJ, USA). The variable heavy domain was PCR amplified from the plasmid provided by Genscript with primers that introduced flanking NcoI and NotI sites; the light chain was similarly amplified to introduce flanking BamH1 and XhoI sites. The PCR products were digested and gel purified prior to ligation into a pET22b derivative containing the linker sequence AAAGSGSGGGSSGGGSSGGGSGASGS, between the NotI (coded by AAA) and the Bam H1 (coded by the C-terminal GS) sites. Similarly, the sequence for the variable heavy and light chain of the anti-DA scFv DA24cB7, joined by a 15 amino acid linker, was synthesized with flanking NcoI and XhoI sites (GenScript) and cloned into the pET22b expression vector.

The anti-STX scFvs and the anti-DA scFv were grown and produced essentially as described previously [48]. Cultures were grown at 25 or 30 °C in terrific broth (TB). Fifty mL overnight cultures were used to inoculate 500 mL of TB and were grown for 3 h before induction by addition of 1 mM isopropyl β-d-1-thiogalactopyranoside (IPTG). After induction, cultures were grown 3 h before pelleting and subjected to an osmotic shock protocol [44,45]. The scFv were purified from the shockate by immobilized metal affinity chromatography followed by size exclusion chromatography using a Superdex 75 10/300 GL column (GE Healthcare, Pittsburgh, PA, USA) and a BioLogic Duo-Flow Chromatography System (Bio-Rad, Hercules, CA, USA). The yield of the scFv was determined by UV spectroscopy, measuring the absorbance at 280 nm using a NanoDrop 2000 (Thermo Fisher, Waltham, MA, USA).

### 4.3. Food Matrices Preparation

A simple sample preparation protocol was used to extract the toxins as compared to the more stringent buffer conditions described in Campbell et al. and Szkola et al. [27,47]. Bay scallops (live, frozen) and live oysters were purchased from a local grocery store. The oysters were placed at −20 °C overnight. The frozen bay scallops (200 g) were blended in a small Cuisinart food processor until smooth with no additional liquid. The puree was placed in 15 mL centrifuge tubes (VWR, Radnor, PA, USA) in 5 g aliquots and frozen until testing. For the frozen oysters, 120.5 g were blended with no additional liquid, aliquoted in 5 g sizes, and frozen until testing.

Just prior to testing, the 5 g samples were thawed and 10 mL PBSTB were added. To the thoroughly blended samples, either STX or DA were spiked to give 1000 ng/mL or 200 ng/mL, respectively. The samples were mixed and incubated for 2 h at room temperature, then spun to remove large particulates. The supernatant was used for analysis. Extraction efficiency was not determined.

### 4.4. Preparation and Biotinylation of mAbs and ScFv

The antibodies were purified from cell supernatants by MEP HyperCel hydrophobic charge induction chromatography (Pall, East Hills, NY, USA), as described previously [49]. Antibodies and scFvs were biotinylated using NHS-LC-LC-Biotin (Thermo-Fischer, Waltham, MA, USA) dissolved in dimethyl sulfoxide (20 g/L). The antibodies were reacted with a 10:1 molar excess of the NHS-LC-LC-Biotin. To enhance the rate of reaction, the pH was increased by the addition of a half-volume of 100 mM sodium borate +100 mM sodium chloride (pH 9.1). After incubation for 1 h at room temperature, the biotinylated antibodies were separated from free biotin by gel filtration on a Bio-gel P10 column (Bio-Rad, Hercules, CA, USA) or by using Zeba spin 7 K desalting columns (Thermo Fisher, Waltham, MA, USA).

### 4.5. Surface Plasmon Resonance Evaluation of Anti-STX mAbs

Surface plasmon resonance (SPR) affinity and kinetics measurements were performed using the ProteOn XPR36 (Bio-Rad). Lanes of a general layer compact (GLC) chip were individually coated with STX covalently linked to an irrelevant HuIgG in 10 mM acetate buffer with pH 5.0. The covalent crosslinking of STX protocol was described previously [15] and in Section 4.6. The STX-HuIgG was attached to the chip following the standard 1-ethyl-3-(3-dimethylaminopropyl)carbodiimide hydrochloride (EDC)/N-hydroxysulfosuccinimide (Sulfo-NHS) coupling chemistry available from the manufacturer. Binding kinetics of each antibody was tested at 25 °C by flowing six concentrations varying from 100 to 0 nM at 100 μL/min for 90 s over the antigen coated chip and then monitoring dissociation for 600 s. For comparison purposes, this data was analyzed using a global Langmuir fit.

### 4.6. Preparation Toxin-Coated MagPlex Microspheres

MagPlex microspheres were coated with DA by first reacting the surface carboxyls with ethylene diamine (EDA) using the standard two step protocol, where 30 µL of the microspheres were washed with 0.1 M sodium phosphate (pH 6.0) three times, and then activated using EDC and Sulfo-NHS 5 mg/mL each. After 20 min the microspheres were washed once with 0.1 M sodium phosphate (pH 6.0) and once with PBS, then microspheres were resuspended in EDA (1 mg/mL) in PBS and incubated overnight. The next day, the microspheres were washed three times with 0.1 M 2-(*N*-morpholino)ethanesulfonic acid buffer (MES) at pH 4.5. The microspheres were then coated with DA by adding a 2:1 EDC:DA ratio. The microspheres were incubated for 1 h and then washed three times with PBS and stored in the dark at 4 °C.

Use of a different chemistry to bind STX to the microspheres was indicated by the immunogen used to prepare the mAbs [15]. MagPlex microspheres (50 µL) coated with STX were prepared by first coating two sets of microspheres with either a purified rabbit or human polyclonal IgG towards irrelevant targets, using the standard protocol described above except they were washed into PBS and then resuspended in 0.4 mL of PBS on the following day. The carbohydrates on the IgG were activated by the addition of 22 µL sodium periodate (46 mM). The microspheres were incubated in the dark for 1 h at room temperature, then washed three times with PBS. The PBS was removed and the microspheres resuspended in 40 µL of 0.1 M sodium bicarbonate (pH 8.5) to which 5 µL of STX (100 µg/mL) was added. The microspheres were incubated for 1 h and then 1 µL of sodium cyanoborohydride (5 M in 1 M NaOH) was added. The reaction was allowed to proceed for 30 min on ice. Then the microspheres were washed three times with PBS and then stored in 0.1 M sodium phosphate (pH 6.0).

### 4.7. Assays

Competitive immunoassay dose response curves were performed first in buffer and then with the spiked food samples. Briefly, added into a well of a 96-well microtiter plate was either a sample containing 1000 ng/mL STX or 200 ng/mL DA such that after serial dilutions (1:4 for STX and 1:3 for DA) 90 µL remained in each well. Next, 10 µL of the biotinylated scFvs (bt-anti-STX, 5F7, at 2.5 µg/mL final for STX and bt-anti-DA at 10 µg/mL final) were added, followed with 10 µL of toxin-coated beads. The foil-covered plate was placed on a FINEPCR micromixer MX4t (Gyeonggi-Do, Korea) for 30 min at room temperature. Using a 96-well flat magnetic plate (BioTek, Winooski, VT, USA), the supernatant was removed and the beads were washed with PBSTB. For signal generation, 50 µL of 2.5 µg/mL SA-PE were added to each well and plate was incubated on shaker for 15 min followed by a wash with PBSTB. Based on previous work which showed a ~5 fold increase in signal [50], a second round of SA-PE was performed as follows. Fifty µL of biotinylated anti-SA (1 µg/mL) were added, incubated for 15 min, and washed with PBSTB. Lastly, another 50 µL of 2.5 µg/mL SA-PE was added and the mixture incubated for 15 min. The beads were washed 2× with PBSTB. PBSTB (100 µL) was added to each well and the plate was analyzed with Luminex MAGPIX. For the assay using the intact mAbs, only a single 30 min incubation with SA-PE (2.5 µg/mL) was performed. Percent inhibition was calculated using the following equation:

100−[(signal/(blank signal))×100]

## Figures and Tables

**Table 1:** Dose response parameters for STX and DA in spiked buffer and food matrices.

	STX			DA		
	Buffer	Oyster	Scallops	Buffer	Oyster	Scallops
Min (%)	3.89 ± 0.92	2.88 ± 0.87	2.37 ± 1.13	3.67 ± 1.09	4.90 ± 2.31	3.43 ± 3.00
Max (%)	89.18 ± 1.37	85.48 ± 1.31	84.65 ± 1.86	87.24 ± 1.36	92.08 ± 2.44	97.44 ± 4.33
IC_10_ (ng/mL)	8.08	9.55	4.43	1.85	1.05	0.94
IC_50_ (ng/mL)	25.57 ± 1.56	31.91 ± 1.75	27.38 ± 2.29	6.91 ± 0.36	4.57 ± 0.47	6.93 ± 1.12
IC_90_ (ng/mL)	93.98	106.85	169.10	25.75	19.96	51.06

**Table 2:** Matrix effects on signal-to-background.

	STX		DA	
	% of Buffer Signal *	Signal/Noise (Max/Min)	% of Buffer Signal *	Signal/Noise (Max/Min)
Buffer	100	7.7 ± 3.4	100	8.8 ± 3.6
Oyster	80 ± 22	8.3 ± 2.2	23 ± 3	12.9 ± 3.2
Bay Scallops	51 ± 11	6.3 ± 1.9	50 ± 23	27 ± 11

Avg and SD of two bead sets from two separate experiments.* ((Max_matrix_ − Min_matrix_)/(Max_buffer_ − Min_buffer_)) × 100.

## Supplementary Materials

The following are available online at www.mdpi.com/2072-6651/8/11/346/s1, Figure S1: Percent inhibition dose response curves for saxitoxin and domoic acid. Figure S2: Dose response curves for mixed saxitoxin and domoic acid assay in buffer.
