# Supplementary material for: Integrating scFv into xMAP Assays for the Detection of Marine Toxins
Source: Toxins (Basel). 2016 Nov 21;8(11):346. doi: 10.3390/toxins8110346 (PMC5127142; doi:10.3390/toxins8110346)
Supplement: Supplementary file 1 [file toxins-08-00346-s001.pdf]

Supplementary Materials: Integrating scFv into xMAP Assays for the Detection of Marine Toxins

Lisa C. Shriver-Lake, Jinny L. Liu, P. Audrey Brozozog Lee, Ellen R. Goldman, Richard Dietrich, Erwin Märtlbauer and George P. Anderson

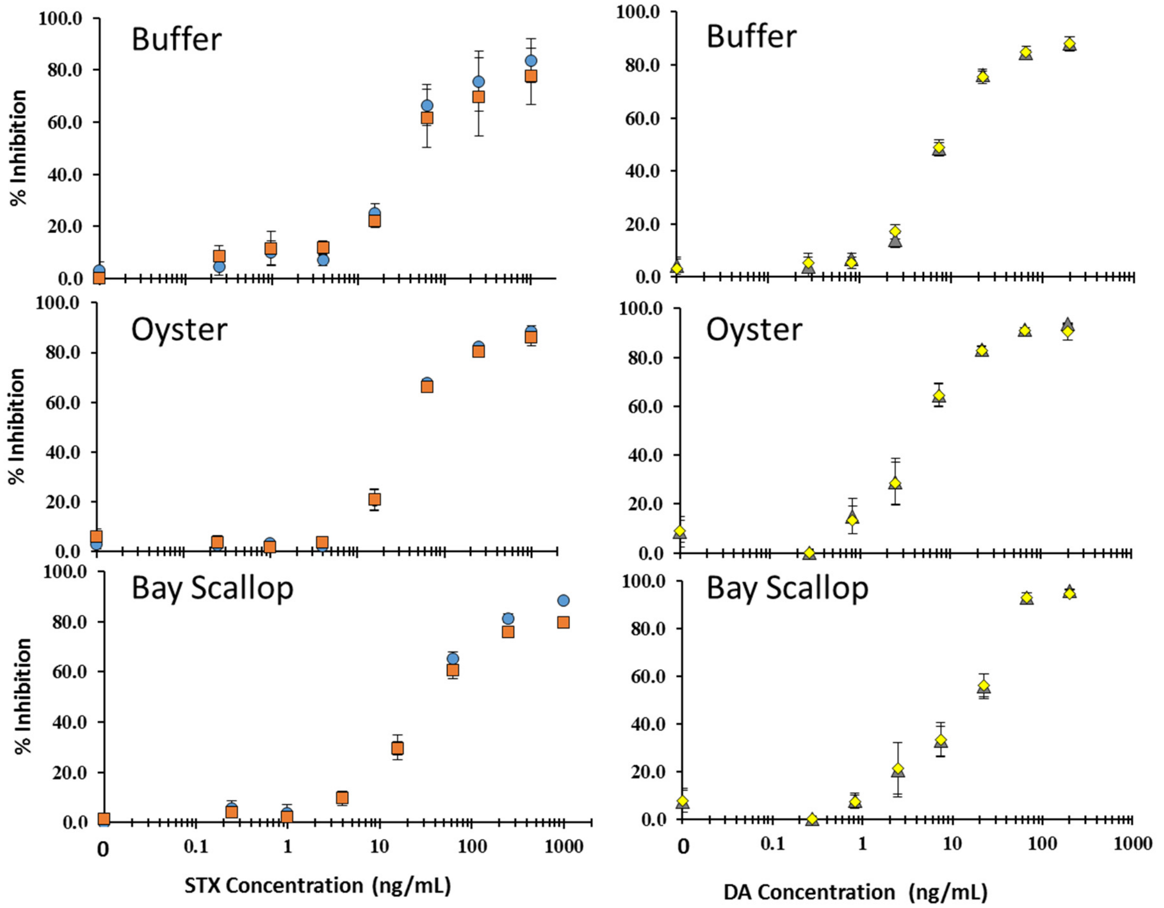

Figure S1. Percent inhibition dose response curves for saxitoxin (STX) and domoic acid (DA). The left side shows STX dose responses in buffer (**top**), oysters (**middle**), and bay scallops (**bottom**). Blue circles—Bead set 26 coated with STX-HuIgG. Orange squares—Bead set 44 coated with STX-RbIgG. The right side shows dose response curves for DA in buffer (**top**), oysters (**middle**), and bay scallops (**bottom**). Both sets of beads have EDA-domoic acid on the surface. The grey triangles represent bead set 54 while the gold stars are for bead set 62. Each point is the average of three experiments plus SEMs.

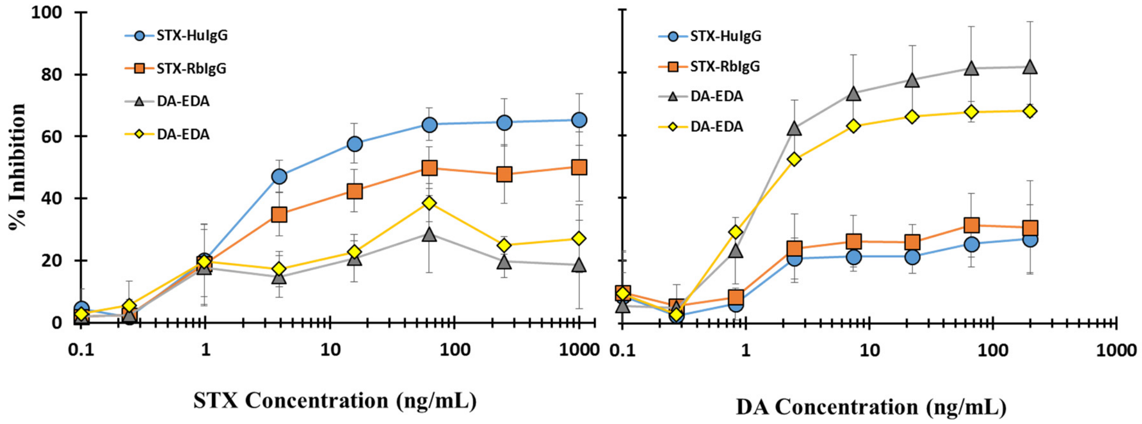

Figure S2. Dose response curves for mixed saxitoxin and domoic acid assay in buffer. The competitive immunoassay contained both STX and DA—coated beads and biotinylated-scFvs for both. Either STX or DA was spiked into the buffer. The blue circles and the red squares are beads specific for STX. The yellow diamonds and grey triangles are for beads specific for DA.
